# Metal-Dependent Adsorption Mechanism in Perfluorinated MIL-140A Metal–Organic Frameworks

**DOI:** 10.1021/acsomega.6c02924

**Published:** 2026-06-08

**Authors:** Francesca Nerli, Francesca Nardelli, Virginia Guiotto, Valentina Crocellà, Marco Taddei, Lucia Calucci

**Affiliations:** † Dipartimento di Chimica e Chimica Industriale, Unità di Ricerca INSTM, Università di Pisa, Via G. Moruzzi 13, 56124 Pisa, Italy; ‡ Istituto di Chimica dei Composti OrganoMetallici—ICCOM, Consiglio Nazionale delle Ricerche—CNR, Via G. Moruzzi 1, 56124 Pisa, Italy; § Dipartimento di Chimica, Centro di Riferimento NIS, Unità di Ricerca INSTM, Università degli Studi di Torino, Via G. Quarello 15/A and Via P. Giuria 7, 10125 Torino, Italy; ∥ Centro per l’Integrazione della Strumentazione Scientifica dell’Università di Pisa, CISUP, Lungarno Pacinotti 43/44, 56126 Pisa, Italy

## Abstract

Perfluorinated metal–organic frameworks (MOFs) with MIL-140A topology display metal-dependent adsorption behavior: while the Ce^IV^ analogue [F4_MIL-140A­(Ce)] exhibits step-shaped CO_2_ adsorption isotherms and strong affinity for H_2_O, both associated with a concerted structural rearrangement of the linkers, the Zr^IV^ counterpart [F4_MIL-140A­(Zr)] shows a classical type I CO_2_ isotherm with a lower adsorption capacity and only weak affinity for H_2_O. A multitechnique approach, including solid-state nuclear magnetic resonance spectroscopy, in situ infrared spectroscopy, and variable temperature powder X-ray diffraction, provided clear evidence that this contrasting behavior arises from the absence of an open metal site on Zr^IV^ and the inability of Zr^IV^ to adopt an octacoordinated geometry, which prevent the establishment of strong metal-guest interactions.

## Graphical abstract

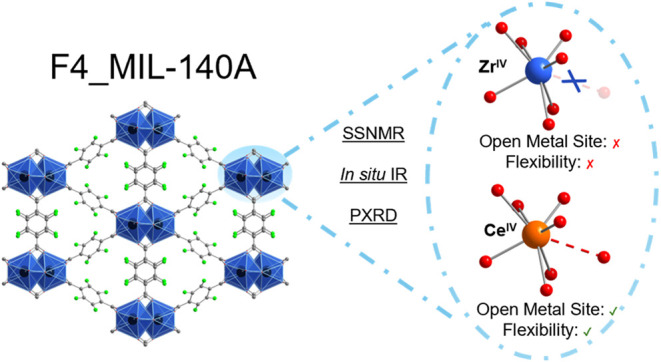

## Introduction

Flexible metal–organic frameworks (MOFs) represent a subclass of soft-porous crystals in which a crystalline organic–inorganic network, composed of metal clusters/ions and organic linkers, self-assembles into a porous architecture capable of undergoing structural transformations upon external stimuli (i.e. temperature, pressure, gas adsorption/desorption).^1,2^

One of the most extensively studied forms of flexibility in MOFs is breathing, a cooperative phenomenon underpinned by a structural transition between a narrow-pore (*np*) and a large-pore (*lp*) phase, often accompanied by a significant change in the unit cell volume, triggered by water adsorption.^1^ Although a well-established model explaining the existence of such phenomenon is still lacking, the pioneering work on the MIL-53 family [M^III^(OH)­BDC, where BDC = benzenedicarboxylate and M^III^ = Al,^3^ Sc,^4^ Cr,^5^ Fe,^6^ Ga,^3^ In] identified the structural requirements for breathing,^1^ and highlighted the key role of the metal center in the framework ability to undergo structural transformations. MIL-53­(Al), MIL-53­(Cr), and MIL-53­(Ga) expand to the *lp* phase upon activation and contract to the *np* phase when exposed to ambient moisture. MIL-53­(Sc) undergoes pore contraction upon activation, while MIL-53­(Fe) remains unchanged, maintaining a constant cell volume. Interestingly, the activated forms of both MIL-53­(Sc) and MIL-53­(Fe) adopt a more compact structure than the typical *np* phase. Notably, the activated phase of MIL-53­(In) has yet to be reported.

The extent to which the metal governs the breathing behavior of the DUT-8 family {M_^II^2_(2,6-NDC)_2_(DABCO), where 2,6-NDC = 2,6-naphthalene dicarboxylate,DABCO = 1,4-diazabicyclo-[2.2.2]-octane and M^II^ = Co, Ni,Cu, Zn} in response to solvent removal, as well as CO_2_ and N_2_ adsorption, was investigated too.^7^ DUT-8­(Ni) undergoes a reversible pore opening upon solvent removal, resolvation, and adsorption of CO_2_ and N_2_. The DUT-8­(Co) congener shows analogous behavior, whereas in DUT-8­(Cu) the framework dynamics is completely switched off upon both solvent removal and gas adsorption. In contrast, DUT-8­(Zn) exhibits an irreversible phase transition upon activation and does not exhibit flexibility in response to CO_2_ and N_2_ exposure.^7^

Given the crucial interplay between the metal center and the structural transformability of breathing MOFs, targeted modifications of the inorganic node may offer a powerful strategy to investigate the metal role also in MOFs displaying other forms of flexibility, such as those undergoing adsorption-induced structural rearrangements involving the concerted rotation of organic linkers, with little or no effect on the unit cell volume.^8–10^ Building on this, in the present work we elucidate the role of the metal in the peculiar behavior observed upon CO_2_ adsorption for F4_MIL-140A­(Ce) [CeO­(F4-BDC)·H_2_O, where F4-BDC = tetrafluoroterephthalate; Figure 1],^11,12^ but not for F4_MIL-140A­(Zr).^13^

**Figure 1:**
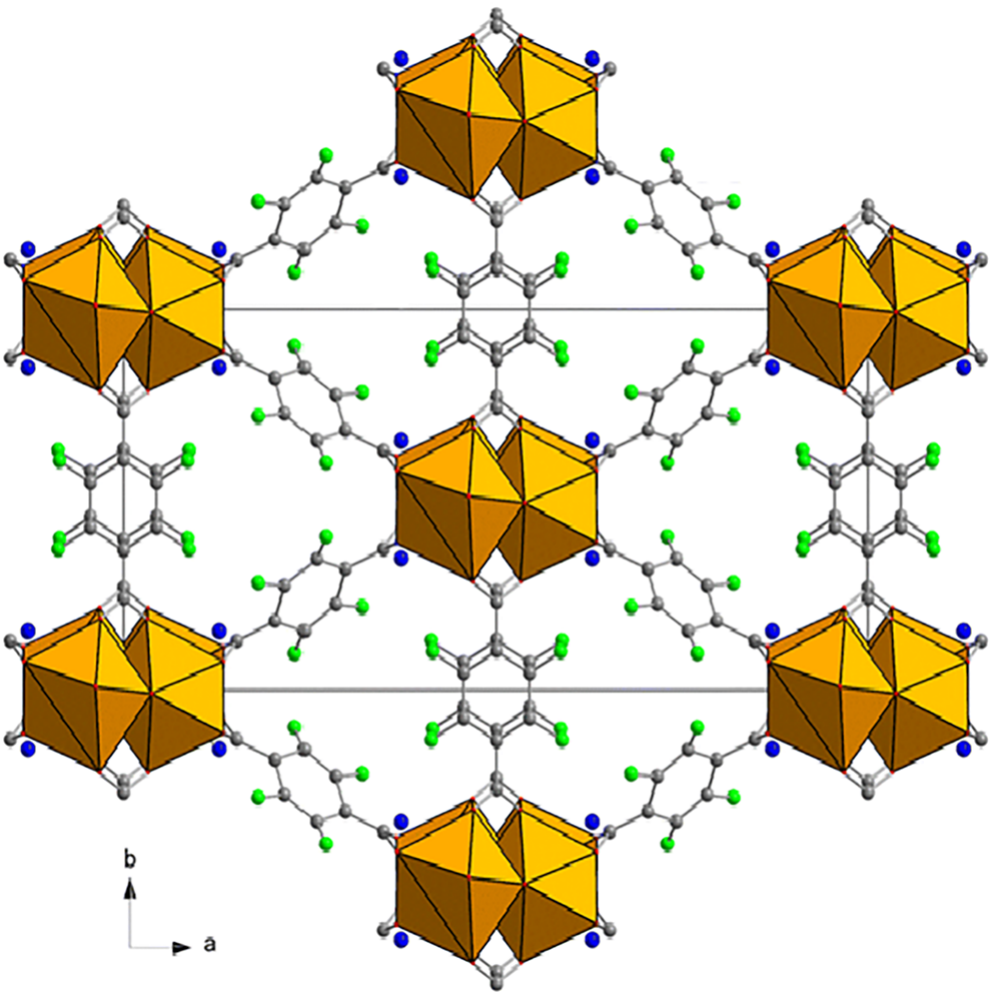
Crystal structure of as synthesized F4_MIL-140A­(Ce). Color code: Ce, yellow; O, red; C gray; F, green; water, blue.

In a study combining powder X-ray diffraction (PXRD), Fourier transform infrared (FTIR) spectroscopy, and quantum mechanical calculations, Zhang et al.^13^ ascribed the CO_2_ adsorption properties of F4_MIL-140A­(Ce) to specific interactions with the electron rich portion of the pore surface created by charge transfer from the ligand to Ce^IV^ ions with low-lying unoccupied 4*f* orbitals, facilitated by the functionalization of the ligands with electron withdrawing fluorine atoms. The step-shaped CO_2_ adsorption isotherms observed for F4_MIL-140A­(Ce) were associated with rotations of benzene rings, whereas no linker rotation, leading to canonical Langmuir type CO_2_ adsorption isotherms, was reported for F4_MIL-140A­(Zr).

An alternative explanation for the step-shaped CO_2_ adsorption isotherms of F4_MIL-140A­(Ce), in which the interaction between CO_2_ and Lewis acidic Ce^IV^ sites plays a key role, was recently proposed by some of us.^11,14^ Open Ce^IV^ sites are generated upon removal of coordinated water from the as-synthesized MOF, where the loss of hydrogen-bond-like interactions between framework fluorine atoms and H_2_O induces a concerted rotation of the phenyl rings (Figure S1a).^11^ A similar phase transition occurs upon CO_2_ adsorption, in which the phenyl rings rotate cooperatively to adopt a conformation that optimizes the interaction between CO_2_ and Ce^IV^ sites (Figure S1b). The binding energy between CO_2_ and the Ce^IV^ sites was determined to be 55.2 kJ mol^–1^ by density functional theory (DFT) calculations, much higher than that predicted for CO_2_ interacting with the linkers inside the channel (35.2 kJ mol^–1^).^11^ The latter value is in line with that found by Zhang et al. for F4_MIL-140A­(Zr) (33.1 kJ mol^–1^), assuming a similar adsorption site.^13^

A subsequent ^13^C solid-state NMR (SSNMR) study demonstrated that CO_2_ in F4_MIL-140A­(Ce) undergoes translational hopping among Ce^IV^ sites consistent with the formation of a Ce–CO_2_ adduct.^14^ More recently, we also demonstrated that linker perfluorination is a critical structural requirement for the CO_2_-induced flexibility.^15^ Indeed, the step-shaped CO_2_ adsorption isotherm and the associated structural rearrangement are no longer observed for F3_MIL-140A­(Ce) and *p*F2_MIL-140A­(Ce), since fluorine removal reduces the steric hindrance of the linker, thereby modifying its ability to adopt an out-of-plane conformation with respect to the dicarboxylate axis and hampering specific interactions with CO_2_.

Whether the flexibility of F4_MIL-140A is intrinsically metal-dependent remains an open question, particularly in relation to the cerium coordination environment and its ability to generate an open metal site (OMS) upon dehydration. Substitution of Ce^IV^ with Zr^IV^, an isoelectronic cation with a smaller ionic radius (0.97 Å for Ce^IV^ vs 0.84 Å for Zr^IV^),^16^ provides a means to elucidate the role of the metal in the flexibility of F4_MIL-140A­(Ce) and to gain further insight into the structural factors governing this behavior. To this aim, F4_MIL-140A­(Zr) was synthesized and a combination of characterization techniques, including PXRD, SSNMR and in situ IR spectroscopies, were employed to probe the molecular origin of the metal-dependent adsorption mechanism.

## Materials and Methods

### Materials

^13^C-labeled (99.0 atom %) carbon dioxide (^13^CO_2_), tetrafluoroterephthalic acid (F4–H_2_BDC), zirconium tetrachloride (ZrCl_4_), acetonitrile (ACN) and acetone were purchased from Sigma-Aldrich.

## Methods

### Synthetic Procedures

F4_MIL-140A­(Zr) samples were prepared in acetonitrile using the linker (F4–H_2_BDC) and zirconium tetrachloride (ZrCl_4_) under varying synthetic conditions, including different molar amounts, stoichiometric ratios, and temperatures. Details of the synthetic screening are reported in the Supporting Information, Section S2.

### Powder X-ray Diffraction (PXRD)

PXRD patterns were collected in the 5–30 °2θ range using a step size of 0.02 °2θ and a scanning rate of 10.0 °2θ min^–1^ with a Rigaku MiniFlex 600-C diffractometer working in Bragg–Brentano geometry and equipped with a D/teX detector, using Cu Kα radiation (1.54056 Å). The X-ray tube was operated at a voltage of 40 kV and a current of 15 mA. Variable temperature PXRD (VT-PXRD) patterns were collected using an Anton Paar BTS-500 chamber. Samples were heated at 40 °C, 80 °C, 120 °C, 160 °C, and 200 °C with a ramp rate of 5 °C min^–1^ and held at each temperature for 10 min to allow for equilibration prior to pattern collection. A pattern was also collected after the heating ramp, when the temperature of the chamber had returned to ambient value.

### Thermogravimetric Analysis (TGA)

TGA was performed in air with a Perkin Elmer Pyris instrument using 5 mg of sample, with a heating rate of 5 °C min^–1^ up to 700 °C.

### Solid-State Nuclear Magnetic Resonance (SSNMR) Spectroscopy

SSNMR experiments were carried out on a Bruker Avance NEO 500 spectrometer working at the ^19^F and ^13^C Larmor frequencies of 470.59 and 125.77 MHz, respectively, equipped with a 4 mm double-channel (H/F-X) CP/MAS probe. 90° pulse duration was 3.15 and 3.90 μs for ^19^F and ^13^C, respectively.

^19^F spectra were recorded using Direct Excitation (DE) Magic Angle Spinning (MAS) experiments at a spinning frequency of 15 kHz, accumulating 64 scans with a recycle delay between consecutive transients of 2–10 s.

^19^F–^13^C cross-polarization (CP) MAS experiments were recorded under high power ^19^F decoupling using a recycle delay of 1–5 s, a contact time of 2 ms, and accumulating 1200 transients for F4_MIL-140A­(Zr) in its activated, hydrated and CO_2_-loaded forms. ^13^C DE MAS spectra were recorded on ^13^CO_2_-loaded samples using a recycle delay of 1–2 s; 100–256 scans were accumulated. All ^13^C MAS experiments were recorded at a spinning frequency of 15 kHz.

^13^C DE spectra under static conditions were recorded on the ^13^CO_2_-loaded sample using a recycle delay of 1 s, optimized to obtain quantitative results for the CO_2_ signal, and accumulating 256 scans.

The chemical shift scale of all nuclei was referenced to the ^13^C signal of adamantane resonating at 38.48 ppm, using the unified scale recommended by IUPAC.^17^

The evacuated and ^13^CO_2_-loaded samples for SSNMR measurements were prepared using a homemade cell provided with a mechanical lever operated from outside enabling the capping of the rotor without disturbing the cell atmosphere. For the evacuated sample, MOF powder, packed into the NMR rotor (4 mm external diameter), was evacuated inside the cell by heating 6 h under vacuum (0.02 mbar) at the temperature of 120 °C and then sealed. For the preparation of the ^13^CO_2_-loaded sample, the cell containing the evacuated sample was loaded with ^13^CO_2_ at 1 bar pressure at RT and the rotor was capped after equilibration for 6 h under the gas atmosphere.

### Infrared (IR) spectroscopy

ATR-IR spectra were recorded with an Agilent Cary 630 FTIR spectrometer equipped with a ZnSe crystal. Each spectrum was recorded at 25 °C within the 4000-648 cm^-1^ spectral range, with a resolution of 4 cm^-1^ and accumulating 16 scans.

In situ IR spectroscopy measurements were performed within the 5000–500 cm^–1^ spectral range using a Bruker Vertex 70 spectrophotometer equipped with a MCT (mercury cadmium tellurium) cryogenic detector. The resolution of the reported IR spectra is 2.0 cm^–1^ and an average of 32 scans was used to enhance the signal-to-noise ratio. Before the analysis, the sample, in the form of a self-supported pellet mechanically protected by a gold envelope, was inserted into a homemade quartz cell with KBr windows. For in situ CO adsorption experiments carried out at cryogenic temperatures, a special IR cell that can be filled with liquid nitrogen to lower the temperature of the system at about −170 °C was employed.

### Gas Sorption Analysis

Adsorption/desorption isotherms were collected using a Micromeritics 3FLEX coupled with a Cryotune dewar from 3P Instruments. For Ar adsorption/desorption measurements at −186 °C, about 30 mg of sample was weighed and activated by heating at 120 °C overnight under dynamic vacuum. The BET SSA was evaluated following the Rouquerol consistency criteria in the range 8·10^–3^–8·10^–2^ *p*/*p*_o_.^18^ Pore size distribution (PSD) and cumulative pore volume (CPV) were evaluated by applying the nonlocal density functional theory (NL-DFT) method by using the MicroActive software provided by Micromeritics. For Ar adsorption curve, the kernel of isotherms with the best fit was selected (NLDFT—Argon on Oxides at −186 °C).

CO_2_ isotherms were collected at 0 °C. To keep isothermal conditions, the sample was inserted into a chiller dewar from Micromeritics in which a coolant or heating fluid, connected to a thermostatic bath (JULABO F25), can recirculate.

### Hydration Experiments

Hydration experiments were carried out by letting a weighted amount of activated sample equilibrate in a closed vessel containing a saturated solution of LiCl (11% RH), CH_3_COOK (22% RH), K_2_CO_3_ (44% RH), or NaCl (75% RH) at RT and, then, weighing the hydrated powder.

## Results and Discussion

F4_MIL-140A­(Zr) was initially synthesized according to the procedure reported by Zhang et al.^13^ (Table S1 and Figure S2). However, the CO_2_ adsorption isotherm obtained at 0 °C for the resulting material, hereafter referred to as F4_MIL-140A­(Zr)_ref, differs markedly from that reported in the original work [hereafter F4_MIL-140A­(Zr)_lit], as shown in Figure S3. This discrepancy prompted a systematic investigation of the effect of synthesis parameters (including temperature, stoichiometry, reaction time, and precursors concentration) on the CO_2_ uptake. All tested synthetic conditions (Table S1) yielded phase-pure MIL-140A materials, with the F4_MIL-140A­(Zr)_D sample exhibiting the highest crystallinity, as evidenced by the sharpness of the Bragg reflections in the PXRD pattern (Figure S2). As shown in Figure S3, F4_MIL-140A­(Zr)_A, F4_MIL-140A­(Zr)_D, and F4_MIL-140A­(Zr)_ref display Langmuir type I adsorption isotherms with comparable CO_2_ uptake values at 1.1 bar (ranging between 0.42 to 0.75 mmol g^–1^), consistently lower than that reported for F4_MIL-140A­(Zr)_lit (∼2.5 mmol g^–1^). These results exclude any significant effect of the synthetic procedure on the CO_2_ adsorption properties of F4_MIL-140A­(Zr). Moreover, the observed uptake values are consistent with those reported for other MIL-140A­(Zr) analogues.^19^ Chemical analyses of all synthesized samples are in agreement with the theoretical composition of F4_MIL-140A­(Zr), based on the derived chemical formula ZrO­(F4-BDC)·H_2_O (Table S2), allowing us to confidently rule out the presence of structural defects or trapped guest molecules that could account for the reduced CO_2_ uptake. Thanks to its higher crystallinity, F4_MIL-140A­(Zr)_D, hereafter simply indicated as F4_MIL-140A­(Zr), was selected for further investigations.

We first characterized the textural properties of F4_MIL-140A­(Zr) in comparison with those reported for F4_MIL-140A­(Ce),^11^ using Ar at −186 °C. As suggested by the type I isotherms^20^ shown in Figure 2a, the textural properties of the two MOFs are quite similar, indicating that the different CO_2_ adsorption behavior of F4_MIL-140A­(Zr) with respect to F4_MIL-140A­(Ce) (Figure 2b) does not originate from differences in the accessible porosity, but instead arises from metal-dependent specific interactions between the framework and CO_2_. A slightly higher BET surface area (252 ± 1 vs 214 ± 1 m^2^ g^–1^) and marginally larger cumulative pore volume and pore size values (Figure S4 and Table S3) were determined for the Zr^IV^-based framework compared to its Ce^IV^-based analogue.

**Figure 2:**
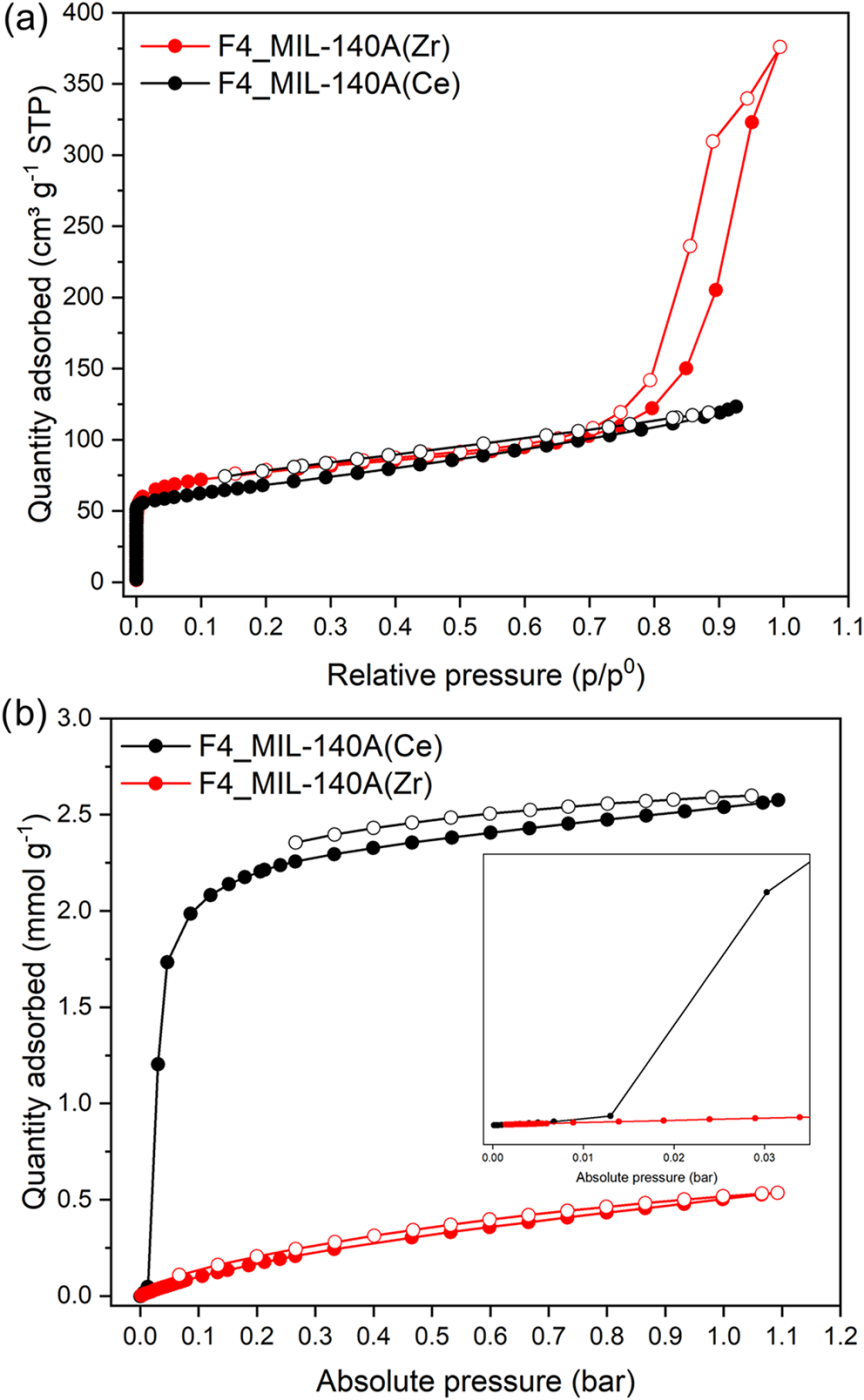
Adsorption (filled symbols) and desorption (empty symbols) isotherms of: (a) Ar at −186 °C and (b) CO_2_ at 0 °C, with a zoom-in of the low-pressure range collected for F4_MIL-140A­(Zr) (red) and F4_MIL-140A­(Ce) (black). Lines are a guide to the eye, not a fitting. The data reported for F4_MIL-140A­(Ce) were previously published in ref 11. and are presented here for comparison with the isoreticular Zr-based sample.

Since the CO_2_-induced flexibility observed in F4_MIL-140A­(Ce) has been associated with the presence of Ce^IV^ OMSs formed upon removal of coordinated water from the as synthesized MOF, we investigated if a similar dehydration process occurs in the Zr-based analogue. Thermogravimetric analysis (TGA) on as-synthesized F4_MIL-140A­(Zr) shows an initial mass loss (5.0%) at 48 °C, likely due to the release of weakly physisorbed water, and a major mass loss of 60.9% at 434 °C, associated with framework decomposition and formation of ZrO_2_, accounting for 34.9% of the initial mass (Figure 3a). No evidence of coordinated water is observed, as indicated by the absence of mass losses at higher temperatures, associated with water desorption from the isoreticular F4_MIL-140A­(Ce), as shown in Figure S5.^12^ TGA data of F4_MIL-140A­(Zr) are consistent with the formula ZrO­(F4-BDC)·H_2_O.

**Figure 3:**
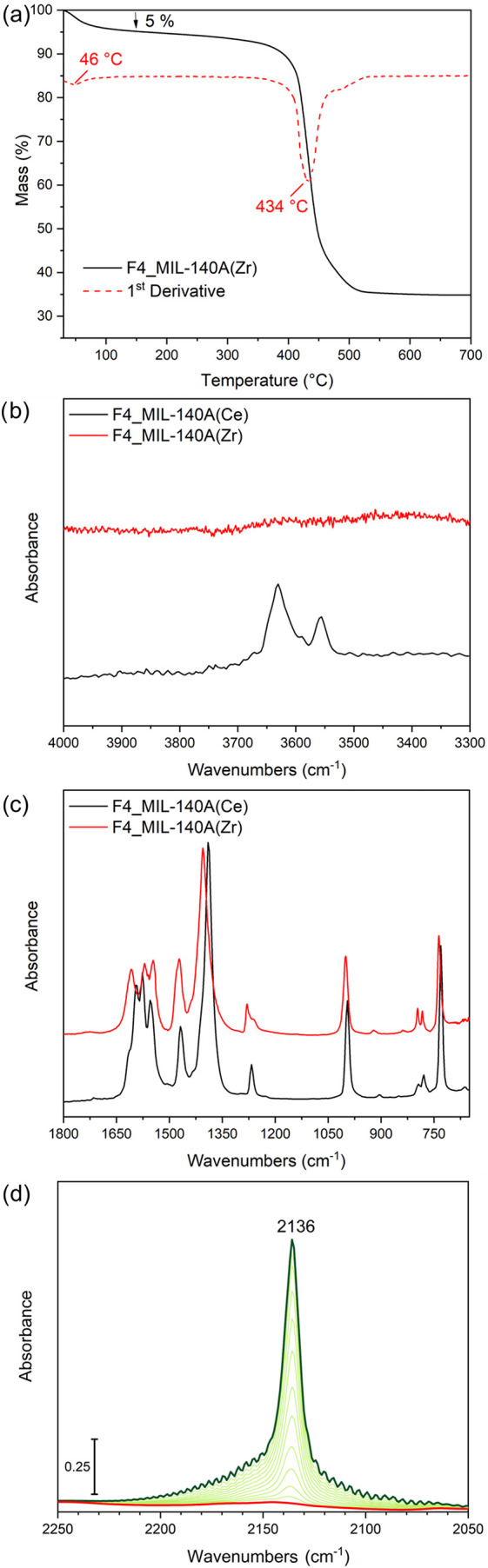
(a) TG curve (black) and 1^st^ order derivative curve (red) of as-synthesized F4_MIL-140A­(Zr). Zoom-in of ATR-IR spectra of F4_MIL-140A­(Zr) (red) and F4_MIL-140A­(Ce) (black) in the spectral region: (b) 4000–3300 cm^–1^ and (c) 1800–600 cm^–1^. (d) In situ mid-IR spectra during CO adsorption/desorption on F4_MIL-140A­(Zr) at cryogenic temperature. Color code: activated material (red curve), maximum CO coverage (dark green curve), outgassing sequence (light green curves).

Water adsorption was further investigated by gravimetric measurements performed on activated F4_MIL-140A­(Zr) exposed to controlled relative humidity (RH) atmospheres. As reported in Table S4, the sample adsorbs a very small amount of water (0.24 H_2_O molecules per Zr) at 11% RH. The uptake of water gradually increases with RH, reaching 0.73 and 1.25 H_2_O molecules per Zr at 44% and 75% RH, respectively. These values are considerably lower than those measured for F4_MIL-140A­(Ce) (e. g., 1.04 H_2_O molecules per Ce at 11% RH, Table S4), indicating a lower affinity of the Zr-based MOF for water.

Further evidence of the absence of H_2_O coordinated to Zr^IV^ was gained by comparing the ATR-IR spectra of F4_MIL-140A­(Zr) and F4_MIL-140A­(Ce) (Figures 3b,c and S6). F4_MIL-140A­(Zr) does not exhibit the characteristic sharp bands in the 4000–3500 cm^–1^ region (Figure 3b), which are diagnostic of metal-coordinated water representing a distinctive structural feature of the MIL-140A­(Ce) topology.^11,15^ On the other hand, no significant differences are observed between the two materials in the spectral region below 1300 cm^–1^, corresponding to the in-plane and out-of-plane aromatic C–F vibrational modes of the perfluorinated linkers.^15^ A slight shift of both the asymmetric and symmetric stretching vibrations of the carboxylate groups is instead observed for F4_MIL-140A­(Zr) with respect to F4_MIL-140A­(Ce) (approximately 1650–1500 and 1450–1350 cm^–1^, respectively, Figure 3c), likely reflecting differences in the electronic properties and polarization behavior of Zr^IV^ and Ce^IV^.^15^

To definitely confirm that F4_MIL-140A­(Zr) does not feature OMSs upon water removal, the interaction with CO was probed by in situ IR spectroscopy at a nominal temperature of −196 °C. As shown in Figure 3d, dosing 60 mbar of CO results in the appearance of a single, weak band centered at 2136 cm^–1^. Due to the high charge density of Zr^IV^, CO coordination is expected to produce bands at significantly higher wavenumbers (2160–2200 cm^–1^). For instance, a band related to the formation of a Zr^IV^–CO adduct has been reported at 2172 cm^–1^ for UiO-66­(Zr) at low temperature, reflecting the strong interaction of CO with coordinatively unsaturated Zr^IV^ sites and a high binding energy (81 kJ mol^–1^ according to DFT calculations).^21–23^ In F4_MIL-140A­(Ce), the Ce^IV^–CO band was detected at 2155 cm^–1^ and a shift to higher wavenumbers is expected in passing from Ce^IV^ to Zr^IV^. The absence of such features in the spectrum of CO adsorbed on F4_MIL-140A­(Zr) confirms the absence of a Zr^IV^ OMS, in analogy to what observed by Guillerm et al. on MIL-140A­(Zr), the congener of F4_MIL-140A­(Zr) with terephthalate linkers,^24^ where Zr is heptacoordinated and no H_2_O molecules are adsorbed on coordinatively unsaturated sites. The weak band observed at 2136 cm^–1^, which easily vanishes upon evacuation, is ascribed to liquid-like CO physisorbed within the channels of F4_MIL-140A­(Zr).^23^ A similar band was previously reported for F4_MIL-140A­(Ce).^11^

To assess possible structural changes of F4_MIL-140A­(Zr) upon dehydration, variable temperature (VT) PXRD experiments were performed by heating the as-synthesized sample from 40 to 200 °C.^15^ The resulting diffraction patterns (Figure S7) show no shifts in the Bragg reflections, indicating the absence of structural transitions of F4_MIL-140A­(Zr) upon activation, in contrast to F4_MIL-140A­(Ce), for which shifts toward higher angles were reported in the temperature range of 160–200 °C (Figure S8a,b).^15^ VT-PXRD measurements provide the first direct evidence of the decisive role played by the metal center in governing the structural transformability of the perfluorinated MIL-140A framework.

The lack of a structural rearrangement of the F4_MIL-140A­(Zr) framework upon hydration/dehydration was corroborated at the atomic level by multinuclear high resolution SSNMR spectroscopy.^25–29^ The ^19^F direct excitation (DE) MAS spectrum of the as synthesized sample (Figure 4) shows two isotropic resonances at −141.8 and −139.9 ppm, indicating the presence of two crystallographically inequivalent linkers in the framework, as also observed for F4_MIL-140A­(Ce)^11,14^ and MIL-140A­(Zr).^30^ Correspondingly, in the ^19^F–^13^C cross-polarization magic angle spinning (CP-MAS) NMR spectrum (Figure 4) each carbon nucleus exhibits signal multiplicity giving rise to resonances at 116.0 and 117.2 ppm (quaternary carbons), 143.4 and 146.4 ppm (fluorinated carbons), and 167.0 and 168.9 ppm (carboxylic carbons). Upon dehydration and subsequent rehydration at different RH values, no changes are observed in either the ^13^C and ^19^F spectra (Figures 4 and S9) indicating that, at variance with F4_MIL-140A­(Ce),^11^ neither conformational changes of the linkers nor removal of hydrogen-bond-like water-linker interactions occur.

**Figure 4:**
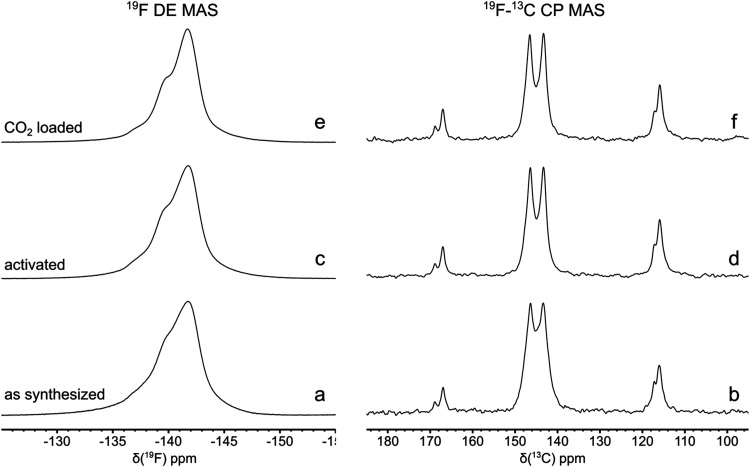
^19^F DE MAS NMR spectra (left panel) and ^19^F–^13^C CP MAS NMR spectra (right panel) of as synthesized (a, b), activated (c, d), and CO_2_-loaded (e, f) F4_MIL-140A­(Zr).

To gain deeper insight into the interactions between CO_2_ and the F4_MIL-140A­(Zr) framework, SSNMR experiments were performed on the ^13^CO_2_-loaded MOF both under MAS and static conditions. As shown in Figure 4, ^13^C and ^19^F MAS NMR spectra of F4_MIL-140A­(Zr) do not change upon CO_2_ loading, indicating no structural changes of the framework, in contrast to what observed for F4_MIL-140A­(Ce).^11,14^ A similar situation was found by Soares et al. in a computational study on MIL-140A­(Zr) loaded with CO_2_.^30^ Calculations showed that MIL-140A­(Zr) does not undergo any significant reorientation of the linkers upon CO_2_ adsorption, and that moderate van der Waals interactions are present between CO_2_ and the pore walls. The signal of ^13^CO_2_ in F4_MIL-140A­(Zr) is selectively detected at 124.7 ppm in ^13^C spectra recorded with DE experiments (Figure 5d–f). In particular, static spectra clearly indicate that CO_2_ undergoes fast isotropic dynamics in the F4_MIL-140A­(Zr) framework, both at 25 and −25 °C (Figure 5e,f), whereas restricted dynamics, i.e., on site wobbling combined with translational hopping among cerium sites on which CO_2_ shows a preferential orientation, was previously found in the F4_MIL-140A­(Ce) framework (Figure 5b,c).^14^ Dynamics of CO_2_ in F4_MIL-140A­(Zr) is more similar to that reported for MIL-140A­(Zr)^31^ in a study combining quasi-elastic neutron scattering and molecular dynamics simulations, which showed that CO_2_ undergoes fast 1D diffusion (mean residence time in each site of 40 ps) along the framework channels, through jumps between areas of the pores located in the vicinity of the organic linkers combined with rotations about the carbon atom. This implies random orientations of CO_2_ at different adsorption sites, in agreement with the isotropic signal observed in the ^13^C static NMR spectra of F4_MIL-140A­(Zr) (Figure 5e,f).

**Figure 5:**
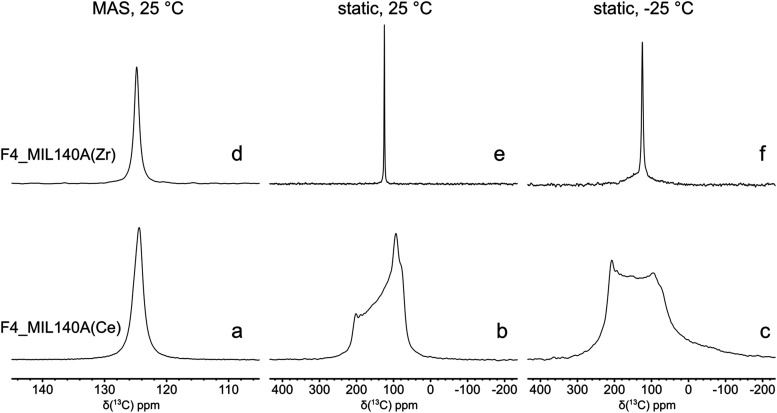
^13^C DE MAS NMR spectra (left panel) and ^13^C DE static NMR spectra (central and right panels) of ^13^CO_2_-loaded F4_MIL-140A­(Ce) (a–c) and F4_MIL-140A­(Zr) (d–f) at the indicated temperatures. MAS spectra were recorded with a spinning frequency of 15 kHz. Spectra of CO_2_ in F4_MIL-140A­(Ce) were already presented in ref 14.

Overall, the comparison of the SSNMR data on F4_MIL-140­(Zr) and F4_MIL-140A­(Ce)^11,14^ reveals fundamentally different CO_2_ adsorption and dynamics in the two materials. In F4_MIL-140A­(Ce), adsorbed CO_2_ occupies a single environment, where it interacts simultaneously with the Ce^IV^ site and the linkers, and undergoes translational hopping among neighboring sites with a relatively high activation energy (46 kJ mol^–1^). This value is comparable to the heat of CO_2_ adsorption for this MOF,^14^ suggesting that CO_2_ dynamics requires disengagement from the Ce^IV^ OMS and the cooperative motion of linkers. In contrast, fast isotropic dynamics is observed for CO_2_ in F4_MIL-140A­(Zr), even at low temperature, indicating that strong interaction sites are lacking due to the absence of OMS on Zr^IV^ and to weak interactions with the fluorinated linkers.

## Conclusions

In conclusion, we unraveled the central role of the metal node in governing the cooperative flexible behavior of perfluorinated MOFs with MIL-140A topology upon H_2_O and CO_2_ adsorption. The absence of an OMS on Zr^IV^ and the inability of Zr^IV^ to adopt an octacoordinated geometry emerge as key factors that prevent the interaction of these adsorbates with the metal and suppress the associated concerted rotation of the linkers that underpin the flexible behavior of the Ce^IV^ analogue. The insight gathered into the role of the metal in such mechanism complements our recent findings on the importance of the degree of fluorination of the linker, providing a comprehensive mechanistic picture of the adsorption behavior of the MIL-140A family of MOFs. Notably, previous computational findings on the CO_2_ binding energies in F4_MIL-140A­(Zr) and F4_MIL-140A­(Ce) are consistent with and support our experimental evidence regarding the nature of the CO_2_ adsorption sites within the perfluorinated MIL-140A architecture.

## Supplementary Material





## Data Availability

The data underlying this study are openly available in Zenodo at 10.5281/zenodo.20540567.

## Notes

AIF (ZIP)
Crystal structures of activated and CO_2_-loaded F4_MIL-140A­(Ce); screening of the synthetic procedures for the preparation of F4_MIL-140A­(Zr); PXRD, CO_2_ adsorption isotherms and elemental analyses of F4_MIL-140A­(Zr) samples; textural properties of F4_MIL-140A­(Zr); thermogravimetric analyses of F4_MIL-140A­(Zr) and F4_MIL-140A­(Ce); ATR-IR spectra of F4_MIL-140A­(Zr) and F4_MIL-140A­(Ce); VT-PXRD data on F4_MIL-140A­(Zr) and F4_MIL-140A­(Ce); controlled hydration followed by gravimetric measurements and SSNMR experiments (PDF)

## Abbreviations

NMR: nuclear magnetic resonance
SSNMR: solid-state nuclear magnetic resonance
CP: cross-polarization
MAS: magic angle spinning
DE: direct excitation
PXRD: powder X-ray diffraction
VT-PXRD: variable temperature powder X-ray diffraction
ATR-IR: attenuated total reflectance-infrared spectroscopy
