# Supplementary material for: Metal-Dependent Adsorption Mechanism in Perfluorinated MIL-140A Metal–Organic Frameworks
Source: ACS Omega. 2026 Jun 8;11(24):35957–64. doi: 10.1021/acsomega.6c02924 (PMC13294966; doi:10.1021/acsomega.6c02924)
Supplement: Supplementary file 2 [file ao6c02924_si_002.pdf]

Supporting Material for

Metal-dependent Adsorption Mechanism in Perfluorinated MIL-140A Metal-Organic Frameworks

Francesca Nerli,[1] *Francesca Nardelli,*[1,2,4] *Virginia Guiotto,*[3] *Valentina Crocellà,*[3] *Marco Taddei,*[1,4*] *Lucia Calucci*[2,4*]

[1]Dipartimento di Chimica e Chimica Industriale, Unità di Ricerca INSTM, Università di Pisa, Via G. Moruzzi 13, 56124 Pisa, Italy

[2]Istituto di Chimica dei Composti OrganoMetallici - ICCOM, Consiglio Nazionale delle Ricerche – CNR, via G. Moruzzi 1, 56124 Pisa, Italy

[3]Dipartimento di Chimica, Centro di Riferimento NIS, Unità di Ricerca INSTM, Università degli Studi di Torino, Via G. Quarello 15/A and Via P. Giuria 7, 10125 Torino, Italy

[4]Centro per l'Integrazione della Strumentazione Scientifica dell'Università di Pisa – CISUP, Lungarno Pacinotti 43/44, 56126 Pisa, Italy.

S1. Structure of F4_MIL-140A(Ce)

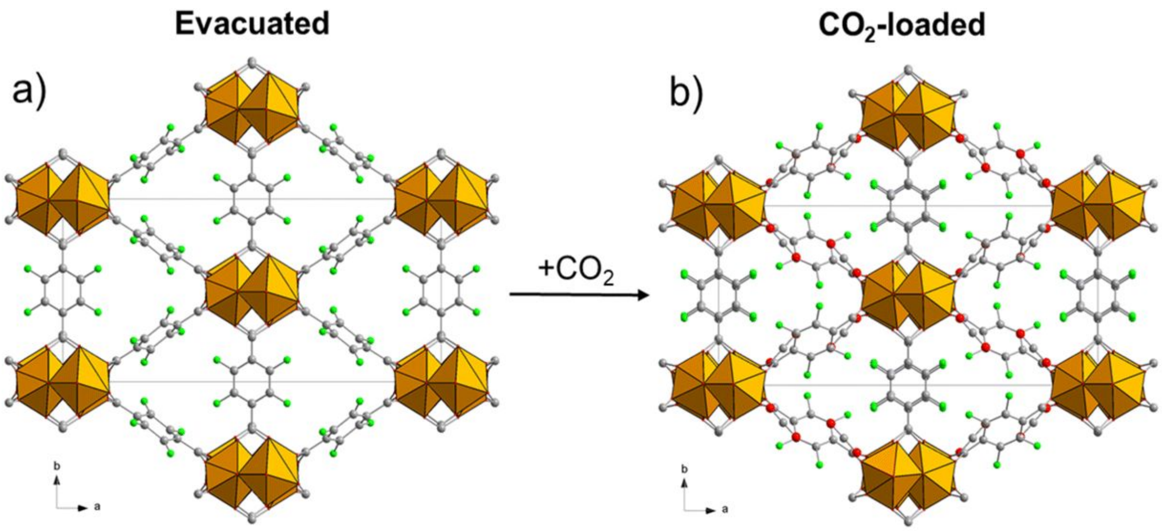

Figure S1. Crystal structures , viewed along the *c* axis, of (a) activated and (b) CO_2-loaded F4_MIL-140A(Ce). By comparing the structures of evacuated and CO_2 loaded F4_MIL-140A(Ce), a cooperative ring rotation can be observed upon CO_2 adsorption.[1]

S2. Synthetic screening and basic characterization

General synthesis procedure

F4_MIL-140A(Zr)_ref, F4_MIL-140A(Zr)_A, F4_MIL-140A(Zr)_B, and F4_MIL-140A(Zr)_C were synthesized following a general procedure. The linker (F4-H_2BDC) and the metal precursor ($ZrCl_4$) were combined in a round-bottom flask in the stoichiometric ratios and molar amounts reported in Table S1. ACN (volume specified in Table S1) was then added, and the reaction mixture was refluxed under magnetic stirring for the indicated reaction time (Table S1).

For the synthesis of F4_MIL-140A(Zr)_D, F4-H_2BDC and $ZrCl_4$ were placed in a Q-tube® equipped with a magnetic stir bar, and 3 mL of ACN was added. The tube was sealed and heated in a preheated aluminum block at 140 ˚C for 2 h under stirring.

In all cases, after completion of the reaction, the obtained mixture was centrifuged and the resulting white solid was washed once with water and twice with acetone, and oven dried at 90 ˚C.

Table S1. Summary of tested synthetic conditions to obtain F4_MIL-140A(Zr) samples.

Sample	F4-H_2BDC	$ZrCl_4$	Linker-to-metal molar ratio	Linker concentration (M)	ACN volume (mL)	Reaction time (h)	Temperature (˚C)	Yield[a] (%)
**ref**	1 mmol, 0.238 g	1 mmol, 0.233 g	1:1	0.02	50	48	120	68
**A**	1 mmol, 0.238 g	1 mmol, 0.233 g	1:1	0.02	50	2	90	85
**B**	5.1 mmol, 1.218 g	5.1 mmol, 1.193 g	1:1	0.11	50	2	90	77
**C**	5.1 mmol, 1.218 g	12.2 mmol, 2.833 g	1:2.37	0.11	50	2	90	60
**D**	0.32 mmol, 0.076g	0.76 mmol, 0.178 g	1:2.37	0.11	3	2	140	45

[a] Calculated assuming the chemical formula ZrO(F4-BDC)

PXRD analysis

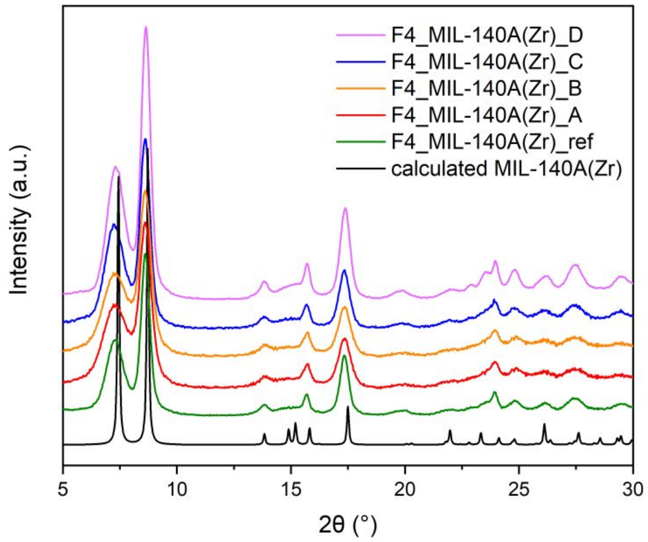

Figure S2. PXRD patterns of the indicated samples compared to the calculated pattern of MIL-140A(Zr) (CSD refcode ZONBAH).

CO_2 adsorption isotherms

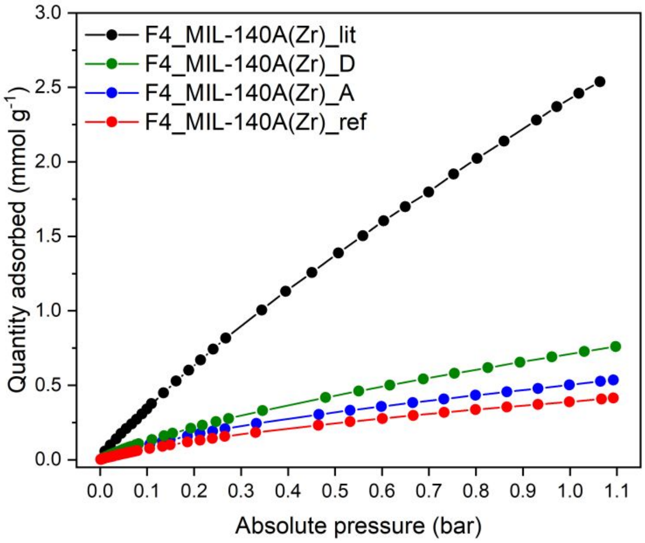

Figure S3. CO_2 adsorption isotherms collected at 0 °C for the indicated samples. The isotherm reported for F4_MIL-140A(Zr)_lit was reproduced from Zhang *et al.*[2] using the *WebPlotDigitizer* software (https://automeris.io/). Lines are provided as a guide to the eye and do not represent fittings.

Elemental analysis

Table S2. Results of C and H elemental analyses performed on as-synthesized F4_MIL-140A(Zr) samples from different synthetic procedures compared with theoretical values calculated for a defect-free F4_MIL-140A(Zr) structure with chemical formula ZrO(F4-BDC)·H_2O.

Element	Theoretical ZrO(F4-BDC)·H_2O	F4_MIL-140A(Zr)_lit	F4_MIL-140A(Zr)_A	F4_MIL-140A(Zr)_B	F4_MIL-140A(Zr)_C	F4_MIL-140A(Zr)_D
**C (%)**	26.59	27.69	26.95	27.16	23.81	26.62
**H (%)**	0.55	0.543	0.84	0.93	0.912	0.719

S3. Textural properties

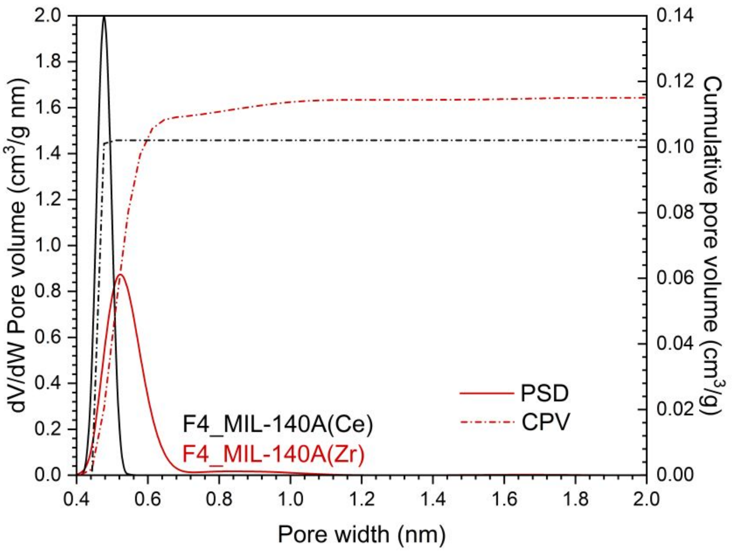

Figure S4. PSD (continuous lines) and CPV (dot-dashed lines) curves of F4_MIL-140A(Ce) (black) and F4_MIL-140A(Zr) (red).

Table S3. Textural properties parameters of F4_MIL-140A(Zr) and F4_MIL-140A(Ce).

Sample	SSA_{BET} (m^2 g^{-1})	CPV (cm^3 g^{-1})	Pore size (nm)
F4_MIL-140A(Zr)	252 ± 1	0.11	0.53
F4_MIL-140A(Ce)[1]	214 ± 1	0.096	0.48

S4. Thermogravimetric analysis

Thermogravimetric (TG) curves (black) and the corresponding first-order derivative curves (red) of as-synthesized F4_MIL-140A(Ce) and F4_MIL-140A(Zr), recorded in the 30-250 °C temperature range, reveal distinct mass-loss behavior. In the case of F4_MIL-140A(Ce), two mass-loss steps are observed, as indicated by two minima in the derivative profile. The first minimum, centered at 36 °C, is attributed to the release of physisorbed water. The second minimum, observed at 110 °C, corresponds to the removal of coordinated water, which requires higher thermal energy for desorption. Notably, this latter feature is absent in the thermogravimetric profile of F4_MIL-140A(Zr), indicating the absence of coordinated water. Indeed, a sole minimum is observed for this sample at 48 °C ascribable to the removal of physisorbed water.

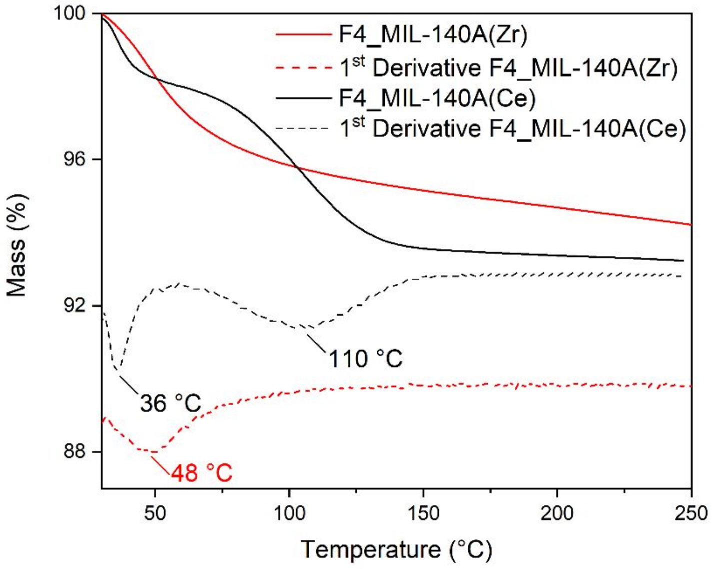

Figure S5. Thermogravimetric (TG) curves (dashed lines) and corresponding first-order derivative curves (dotted lines) of as-synthesized F4_MIL-140A(Zr) (red) and F4_MIL-140A(Ce) (black), recorded over the 30–250 ˚C temperature range.

S5. ATR-IR spectroscopy

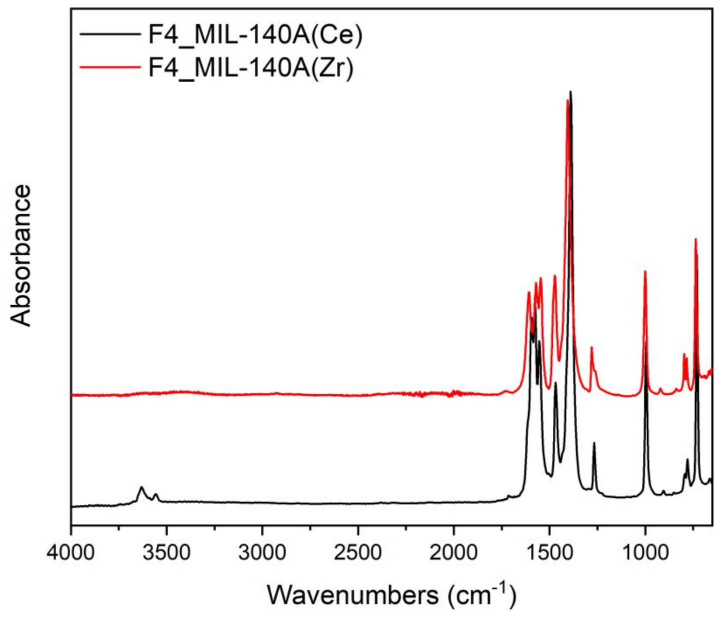

Figure S6. ATR-IR spectra of F4_MIL-140A(Zr) (red) and F4_MIL-140A(Ce) (black).

S6. VT-PXRD analysis

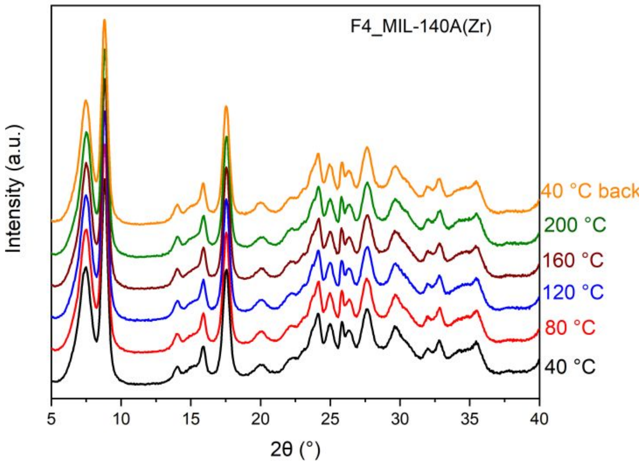

Figure S7. VT-PXRD patterns of F4_MIL-140A(Zr) collected on heating between 40 and 200 ˚C. The pattern marked "40 ˚C back" was collected after cooling down from 200 to 40 ˚C without opening the sample chamber.

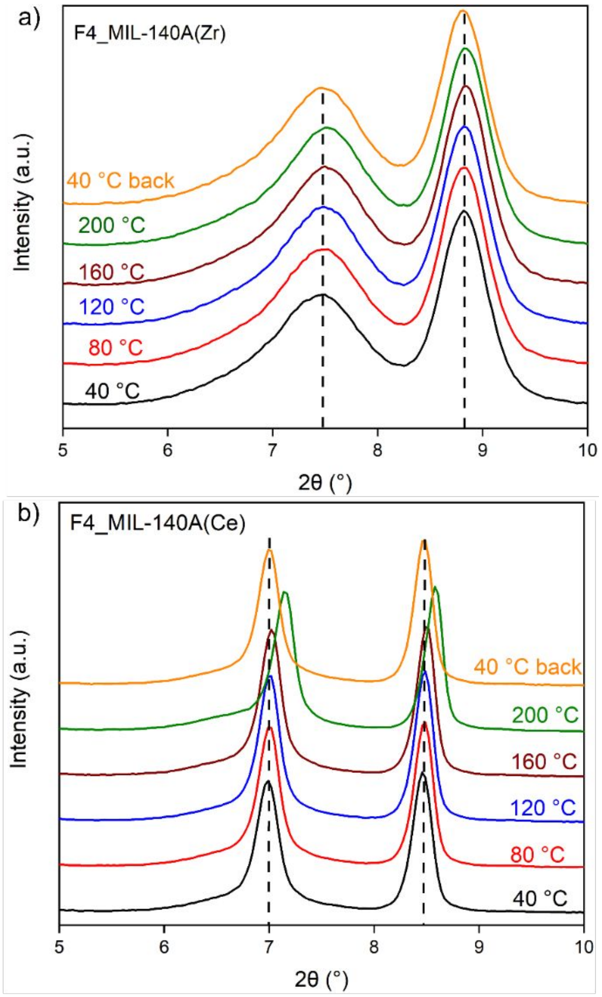

Figure S8. Low-angle region of the VT-PXRD patterns of: (a) F4_MIL-140A(Zr) and (b) F4_MIL-40A(Ce).[3]

S7. Hydration of F4_MIL-140A(Zr)

Table S4. Water adsorbed (molecules of H_2O per metal atom) by F4_MIL-40A(Zr) and F4_MIL-140A(Ce) during hydration experiments under controlled humidity atmosphere.

Sample	11 % RH	44 % RH	75 % RH
**F4_MIL-140A(Zr)**	0.24 H_2O/Zr	0.73 H_2O/Zr	1.25 H_2O/Zr
**F4_MIL-140A(Ce)**	1.04 H_2O/Ce	1.44 H_2O/Ce	1.92 H_2O/Ce

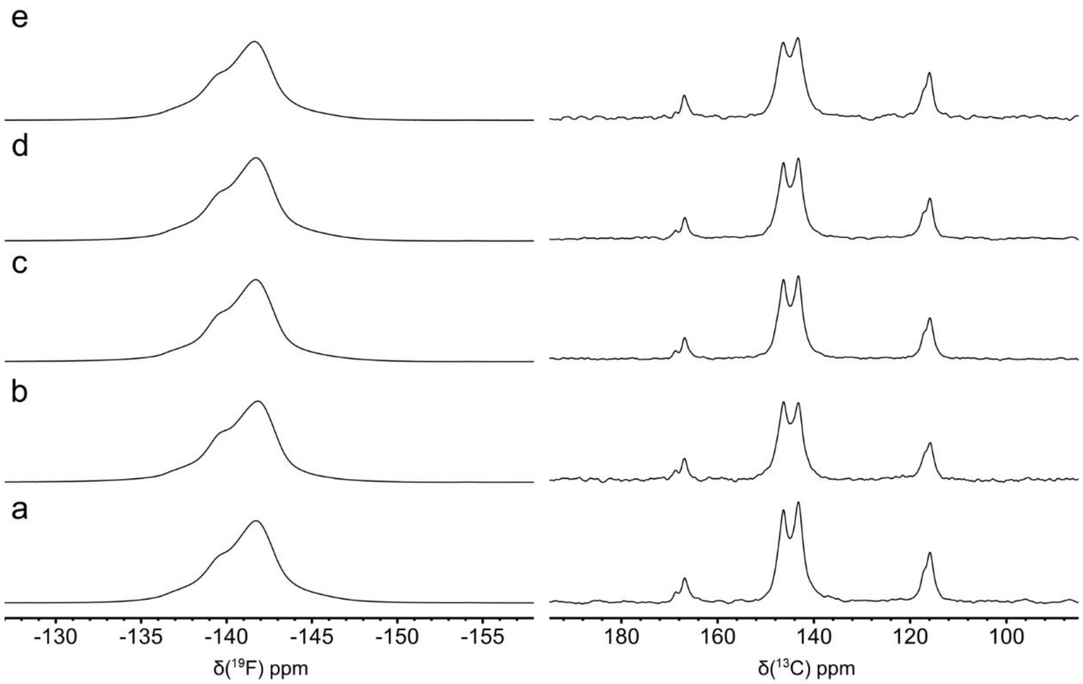

Figure S9. ^{19}F DE MAS NMR (left panel) and ^{19}F-^{13}C CP MAS (right panel) NMR spectra of F4_MIL-140A(Zr): (a) as synthesized; (b) activated; after hydration of the sample at (c) 11 %, (d) 44 % and (e) 75 % RH.

References

(1) Cavallo, M.; Atzori, C.; Signorile, M.; Costantino, F.; Venturi, D. M.; Koutsianos, A.; Lomachenko, K. A.; Calucci, L.; Martini, F.; Giovanelli, A.; Geppi, M.; Crocellà, V.; Taddei, M. Cooperative CO_2 Adsorption Mechanism in a Perfluorinated Ce^{IV} -Based Metal Organic Framework. *J. Mater. Chem. A* **2023**, *11* (11), 5568–5583. https://doi.org/10.1039/D2TA09746J.

(2) Zhang, Z.; Peh, S. B.; Krishna, R.; Kang, C.; Chai, K.; Wang, Y.; Shi, D.; Zhao, D. Optimal Pore Chemistry in an Ultramicroporous Metal–Organic Framework for Benchmark Inverse CO_2 /C_2 H_2 Separation. *Angew. Chem. Int. Ed.* **2021**, *60* (31), 17198–17204. https://doi.org/10.1002/anie.202106769.

(3) Nerli, F.; Guiotto, V.; Nardelli, F.; Giovanelli, A.; Bizzarro, L.; Zizzi, F.; Signorile, M.; Geppi, M.; Calucci, L.; Crocella', V.; Lessi, M.; Taddei, M. On the Role of Linker Fluorination in the Adsorptioninduced Structural Response of CeIV-Based MetalOrganic Frameworks. *Chem.-Eur. J.* https://doi.org/10.1002/chem.70830.
